# Expertise-dependent perceptual performance in chess tasks with varying complexity

**DOI:** 10.3389/fpsyg.2022.986787

**Published:** 2022-11-03

**Authors:** Thomas Küchelmann, Konstantinos Velentzas, Kai Essig, Dirk Koester, Thomas Schack

**Affiliations:** ^1^Department of Neurocognition and Action-Biomechanics, Faculty of Sport Sciences, Bielefeld University, Bielefeld, Germany; ^2^Center of Cognitive Interaction Technology, Bielefeld University, Bielefeld, Germany; ^3^Faculty of Communication and Environment, Rhine-Waal University of Applied Sciences, Kamp-Lintfort, Germany; ^4^Faculty of Sport Sciences and Personality, BSP Business and Law School, Berlin, Germany

**Keywords:** priming, perceptual processing, chunking, prime-target complexity, decision making

## Abstract

Perceptual performance, anticipating opponents' strategies, and judging chess positions especially in subliminal processing is related to expertise level and dependent on chunking processes. It becomes obvious that chess expertise is a multidimensional phenomenon related predominantly to experience. Under consideration of chess expertise categorization, we conducted two priming experiments expanding existing designs by gradually increasing the target and task complexity. The main aim was the evaluation of potential visuocognitive limitations. The results reveal experts' perceptual superiority manifested by their faster reaction times in settings with increased stimulus and task complexity. Further, experts' priming effects seem to be affected by the target content and/ or priming duration. For short prime duration, experts show priming effects only for less complex prime-target content. Interestingly, for longer prime duration and more complex prime-target content, all participants reveal priming effects. In summary, we argue that experts' visuocognitive processing (i.e., detecting or anticipating potential threats to the king) is rooted in a more efficient visuocognition due to stored chunks of checking and mating constellations. We suggest that visuocognitive limitations are related also to the prime-target complexity as well as to the task. Further investigations must be conducted in order to elucidate the factors with an increased impact on chess players' performance.

## Introduction

The term “expertise” is used in various domains and is also part of everyday language. The definition of expertise is mainly related to indicators such as social reputation and completed education. However, in a specific domain expertise is highly correlated with performance perfection and dedicated exercise (Janelle and Hillman, 2003). Specifically, high expertise is attributed with more than 10 years of experience, which primarily refers to mental tasks in a specific domain (Chi et al., 1988; Klein and Hoffman, 1992). Experts should show reproducible predominant performance on representative tasks within their domain as well as in the context of laboratory and standardized tests (Ericsson and Smith, 1991). Under this point of view, cognitive skills such as perception superiority and/ or information processing are indicated to play a crucial role for high-level expertise (Chase and Simon, 1973b; Gobet and Simon, 1996; Charness et al., 2001). Concerning the question of how high performance can be achieved, numerous research studies indicate that in any domain intense practice and varied training is required (Galton, 1979; Ericsson, 1996; Ericsson and Lehmann, 1996). Ackerman and Cianciolo (2000) argue that dedicated practice enables practitioners to improve their cognitive perceptual skills and Schack et al. link those skills with the demonstrated performance (Schack et al., 2014). To summarize the required skills for reaching expertise (i.e., perception, information processing, decision-making, problem solving, etc.) the term of *visuocognition* is established in the literature (e.g., Warren, 1993).

In the last decades, chess has become an outstanding object of research not only in strategic (decision-making) research, but also in the fields of visual searching and analysis. Chess is mentioned as the “*Drosophila of Artificial Intelligence*” (Simon and Chase, 1973; McCarthy, 1990). Moreover, Simon and Chase (1973) recommend chess as a prototype for many expertise domains because of its extremely high state-space complexity. The amount of 10^47^ legal game positions are reachable from the starting position, and this impedes the *decision-making* processes and strategy adaption during the game (Shannon, 1950; Gobet and Charness, 2006). Moreover, the fact that two chess matches are never exactly the same, and players never handle the exact same constellations during a chess match, underlines the complexity chess players have to deal with.

### Background and related work

If the body is the hardware, neurocognitive processing can be seen as the software, which makes the “system” work faster. The performance of this software is higher the more chunks it uses, considering that chunks are clearly arranged small units of significant domain-specific information (Miller, 1956). To understand chess players' visuocognitive processing, the working memory became one of the central concepts. The study of De Groot (1966) represents the origin of research to capture the essence of chess memory skills. Specifically, De Groot (1966) employed recall tasks and think aloud protocols in decision-making during problem solving. In fact, the results show performance difference (correctness of decision) between grand masters and candidate masters in detecting the most efficient move in given chess positions. The think aloud protocols' analysis revealed that both participating groups reported a similar number of moves (e.g., problem solving) but grand masters as the highest skilled players found on the one hand more efficient solutions – chess moves – and at the same time they achieved results more quickly. De Groot's study is criticized due to the small sample size, the statistical analysis, and finally the participants' selection. Nevertheless, it remains a milestone of chess research. In line with de Groot's studies, Chase and Simon (1973a,b) were able to show that chess experts have significantly higher performance than less skilled players when memorizing meaningful chess constellations. Chase and Simon argue that those results can been explained by the chunking theory. At the same time, Simon and Gilmartin (1973) conclude that chunks are composed of five to eight meaningfully related pieces, and they estimate that there are approximately 50,000 chunks stored in the memory of a chess grand master. This calculation was based on the reproduction's analysis of chess game constellations through the participating grand masters. As a conclusion, it becomes obvious that chunks enhance the visuocognitive processing allowing chess experts to anticipate consequences of potential moves during a game and to select the best ones.

Reingold et al. (2001) in a reaction time experiment presented check constellations on a computer screen, whereas one of two pieces was checking the king. One of them was cued depending on the manipulation (attacking or not). The participants should decide as fast as possible if the cued piece checked the king. During the test, the experts showed difficulties inhibiting irrelevant information (e.g., not cued piece) and they only showed an interference effect compared to novices. In contrast, experts showed a faster reaction time when the attacker was cued. This indicates task-specific automaticity and perceptual encoding advantages of chess experts in check detection tasks (static chess situations).

Additionally, Kiesel et al. (2009) conducted a two-settings experiment using subliminal presentations of checking and non-checking constellations in the first setting and simpler but uncommon chess configurations for the second, whereas a single piece was allocated on a black or white square. In the first setting two groups of experts and novices should perform a two-choice response, with respect to target stimuli (check or no-check for the first setting, and black or white for the second) under time pressure. Each target stimulus was preceded by a so-called prime. Those were congruent or incongruent to the target. The authors found out that the experts benefit from the congruent priming, which triggers the task-relevant response (congruent primes preactivate the required response to the target) whereas incongruent primes preactivate the contrary (Dehaene et al., 1998; Eimer and Schlaghecken, 2002). No congruency effects were found for the group of novices in the first setting. In the second setting with the simplified condition, only the experts participated, and they showed no effects. The authors esteem that the priming effects by experts should be related to the chunks. However, it is not clear why they investigated only chess experts and novices ignoring the level of intermediates, which could permit them to provide more detailed results.

Supplementary to the above-mentioned studies, Postal (2012) assessed experts, intermediates, and novices by the replication and extension of the Reingold et al. (2001) design. First, the three groups had to perform a detection task with cued attackers and second a memory task involving attentional priming. In general, the results are in line with those of Reingold et al. (2001). However, the implementation of the intermediates in the design, allows a more differentiated analysis as well as more insights regarding the taxonomy and meaning of the different expertise levels. Interestingly, in the memory task only intermediates benefit from attentional priming, which seems to facilitate the selection of relevant information.

From this point of view, visuocognition can be considered as the “*Holy Grail”* of chess-performance. As an additional method for the analysis of visual strategies, Sheridan and Reingold (2014) employed eye-tracking when participants had to identify the most efficient move in given chess constellations. Their results indicate that highly skilled chess players use more effective visual search strategies (e.g., analysis implying the amount and duration of the fixation as well as points of interest) when they distinguish between task-relevant and task-irrelevant regions of a chessboard. Additionally, not only dealing with static chess situations but also task-planning experiments (dynamic chess situations) assume that perceptual skill is a key aspect of chess expertise. For instance, Sheridan and Reingold (2017) designed a screen-based gaze selection task in which participants had to plan the complex movements of the knight. Participants would decide in which of four presented chess constellations the knight could reach a target square within three moves. Following the same analysis procedure as Sheridan and Reingold (2014) the authors underline that experts show a high visuocognitive efficiency regarding their visual searching strategies, which facilitates their decision-making processing, compared to novices. Interestingly, the middle performance stage of the intermediates was also disregarded here.

Recent research on virtual chess players benefits from the given opportunities to analyze the multidimensional visuocognitive processing of several players and to program real players' computer simulators (Dhou, 2018) based on those data. For the programming, the crucial information regards players' personality, their strategical thinking, fortitude, and weaknesses independent from the opponent's skills. However, the consideration of visuocognitive limitations seems to be also important in terms of virtual chess players' perfection/ personalization but also in order to explicitly train visuospatial skills of real human opponents (i.e., through emulation of specific chess constellations during a virtual game).

### Contributions of this work

The main goal of the present study was to identify visuocognitive performance limits, under special consideration of a) the expertise level and b) the task/target complexity. In order to enable a more detailed analysis, we extend the experimental setting of Kiesel et al. (2009) employing check and mate detection tasks in static as well as in planning the next move settings. Further, we manipulate the complexity expanding the size of the presented stimuli as well as the number of involved pieces. Finally, we consider in our design intermediates aiming to bridge the gap between the different expertise levels providing a more detailed analysis. This potentially contributes to expertise research, e.g., to identify incremental changes between intermediates and experts. In order to reach the determined goals, two experiments were conducted.

## Experiment 1: Subliminal priming

Kiesel et al. (2009) could show in a priming paradigm experiment, that only experts (in contrast to novices) are able to respond significantly faster in the case of congruency between the prime and target stimulus (i.e., both showing either check or no check). This means that features of pieces' identities and locations are linked. However, Kiesel et al. (2009) also found evidence for limitations of chess experts' perceptual superiority. The question arising here is to what extent the diversity of expertise affects the results of the study? From this point of view, further investigations should be conducted in order to analyze perceptual differences between more similar groups regarding their expertise and also with a circumstantial differentiation of the provided stimuli.

### Complexity and response time

The more complex a chess task is the longer the processing time is and naturally the reaction time (*RT*). Hence, complexity can be modified by the pieces' configuration – generally, it is more difficult to mate a king than to merely check him – as well as by the number of pieces employed in a chess constellation. A larger number of pieces requires a larger stimulus, [e.g., 4 × 4 chessboards instead of 3 × 3 chessboards such as employed in Kiesel et al. (2009)].

We hypothesize that in both settings of our first experiment experts will show a congruency effect and have a significantly shorter RT than intermediates, who in turn will have a shorter RT than novices. For novices we do not expect a congruency effect at all.

### Materials and methods

#### Design

**Figure 1:**
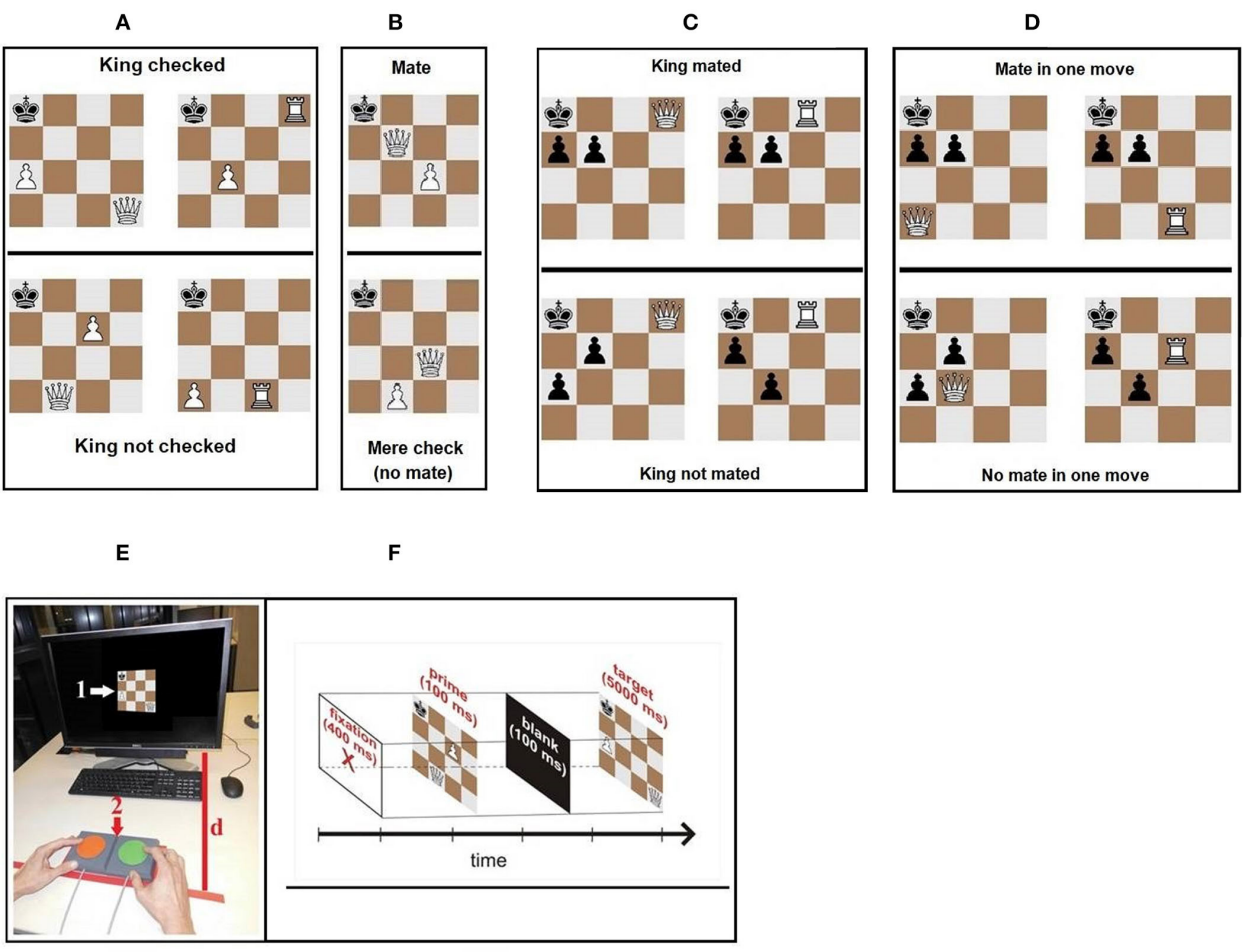
**(A)** Examples for stimuli in Experiment 1, setting 1: check and no check (examples for incongruent prime-target pairs). For each target stimulus combination, the participants have to answer the question: “Is the king checked or not?” **(B)** Examples for stimuli in Experiment 1, setting 2: mate and check (example for an incongruent prime-target pair). For each target stimulus, the participants have to answer the question: “Is the king mated or only checked?” **(C)** Examples for stimuli in Experiment 2, Prime stimuli: a present mate and only a check; **(D)** Examples for stimuli in Experiment 2, Target stimuli: mate and check. For each target stimulus, the participant has to answer the question: “Can the king be mated within the next move?” **(E)** Experimental setup: (1) stimulus presented in the center of the screen; (2) external button box with two buttons; (d) is the distance between the button box and the screen; **(F)** A trial in Experiment 1, Setting 1 (check detection setting): prime (no check) and target (check) are incongruent (prime-target pair condition nc/c).

Experiment 1 of the present study is based on a 3 × 2 design (expertise × prime congruency) having RT as the dependent variable. The type of task determines the difficulty: “a check has to be detected” respectively “a mate has to be detected”. Considering these, we use two different settings with increasing complexity and task difficulty: the *check detection* setting (first setting, see Figure 1A) and the *mate detection* setting (second setting, see Figure 1B).

#### Equipment

In order to realize the present study, one Dell Latitude E6520 Notebook (core 8 with an external HD screen 17inch with 100 Hz) was used. Further, the selected stimuli were provided using the Presentation^®^ software (Neurobehavioral Systems, 2019). Participants' RTs were registered by an external button box (Figure 1E). Participants entered their decision through pushing the green or the red button (the responses “check” vs. “no check” in the *check detection* task and “mate” vs. “mere check” in the *mate detection* task). The distance to the screen was calibrated with the help of a bolstered wood construction (30 cm from the table edge).

#### Participants

A total of 51 chess players (*M*_age_ = 29.61, *SD*_age_ = 12.96) participated in the present study. The first group consists of “experts” (*N*_exp_ = 17, *M*_age_ = 35.53 years, *SD*_age_ = 11.24), the second group of “intermediates” (*N*_int_ = 15, *M*_age_ = 35.27, *SD*_age_ = 13.88) and finally the third group of “novices” (*N*_nov_ = 15, *M*_age_ = 19.84, *SD*_age_ = 6.61). The underlying expertise classification is realized by considering players' ELO or DWZ ratings. From this point of view an ELO/ DWZ score of 1.850 and above qualifies as “expert” and a score between 1.200 and 1.850 for “intermediate”. Participants with an ELO or DWZ below 1.200 were assigned as “novices”. Moreover, players without ELO or DWZ rating who reported to have played a minimum of 20 and a maximum of 100 chess matches in lifetime were also classified as “novices”. Specifically, all novices had to prove they were familiar with the chess rules and the pieces' movement patterns and to be able to distinguish check and mate. All participants completed both experimental settings consecutively in the same order (setting 1 first) and took a break of 15 min in-between. Prior to the data acquisition, participants gave informed consent.

#### Stimuli and procedure

Both settings employ visual chess stimuli on a computer screen conducted as a priming paradigm (Kiesel et al., 2009). All stimuli do not only contain a king and an attacking piece (a queen or a rook) but also in addition a pawn of the same color as the attacker.

For the *check detection* setting, we designed stimuli in which the position of queen, rook, and pawn were never repeated. As revealed by Pohl (2011), a repetition of pieces' positions in unconsciously presented stimuli might cause unintentional location priming effects. For instance, if in one stimulus a queen would check the king and if in the subsequently shown stimulus a rook was on the same square as the queen in the previous stimulus but without check, the form location priming could suggest the rook checks the king.

Accordingly, in the *mate detection* setting the pawn is needed to assist the queen in mating. In order to generate an equal complexity of stimuli in both settings, they both likewise employ a white pawn in each stimulus although the pawn has no influence on checking or non-checking. Moreover, queen and rook have a significantly different form, so the effect of form-priming is excluded (Pohl, 2011). Hence, two pieces with similar form and size (e.g., a bishop and a queen) could cause a priming effect, since they are not clearly distinguishable when they are presented unconsciously. Hence, the situations to be judged are more complex than in Kiesel et al. (2009) namely to distinguish a mere check from a mate. The white queen is either mating or merely checking the king. Since a rook cannot mate the king with the sole assistance of a pawn, rooks are not used in the *mate detection* setting.

Finally, we assumed that the higher complexity of our stimuli would require more intense information processing. Therefore, we use a longer prime presentation time of 100 ms compared to Kiesel et al. (2009) yet staying under the threshold of conscious perception (Elgendi et al., 2018).

All 16 stimuli were 4 × 4 chessboards extending to a 60 mm square. The black king was always located in the upper left corner following the example of Kiesel et al. (2009). According to the two different experimental conditions, the stimuli were subdivided into two categories.

In the *check detection* setting (Figure 1A), four checking and four non-checking positions were used: Two of each kind as targets, respectively as primes. Each contained a white pawn that does not contribute to either checking or non-checking. The attackers were a queen (four stimuli) or a rook (four stimuli) and never occupied the same position. Congruent prime-target pairs had the conditions check/check (*c/c*) or no check/no check (*nc/nc*), incongruent prime-target pairs the conditions check/no check (*c/nc*) and no check/check (*nc/c*), respectively.

In the *mate detection* setting, eight other stimuli were employed showing the white queen assisted by a white pawn. In half of the cases, they were mating the king, in the other half only checking the king. In addition to the *check detection* setting, four stimuli were used as targets (two showing a mate and two showing a check only) and the other four as prime stimuli. The stimuli conditions were referred to as mate (*m*) and check (*c*). Prime-target pairs' conditions were denoted by “*m/m*” or “*c/c*” (congruency) and “*m/c*” or “*c/m*” (incongruity).

Each setting contained 80 trials – every prime-target combination was presented five times in random order. For both settings (e.g., first setting: c/c - nc/c; nc/nc - c/nc; and second setting: m/m - c/m; c/c - m/c), each trial (Figure 1F) starts with a fixation cross in the center of the screen, presented for 400 ms followed by prime target (duration 100 ms) then a blank screen (100 ms) and finally the target (max. 5,000 ms). Participants received no information about the structure of the prime stimuli.

### Results setting 1

We hypothesized that in both settings experts show a significantly shorter RT and congruency effect compared to intermediates which in turn we expected to have a shorter RT than novices. Further, we expected no congruency effect at all for the last two groups.

We took the number of correct answers (*CA*) as proof of performance and performed a one-way ANOVA with the number of the correct responses as a dependent variable. The number of trials was 80. The results revealed no significant differences between the groups. Moreover, we performed, for all group comparisons regarding the different prime-target combinations, one-way ANOVAs with RT as the dependent variable. Only correct responses were considered for all calculations. For an overview of the descriptive statistics (i.e., means, standard deviations, standard error, and percentage of correct answers) see Table 1.

**Table 1:** Overview of the descriptive statistics for all experimental settings.

	**Experiment 1/setting 1**					**Experiment 1/setting 2**					**Experiment 2**				
**Group**	**Prime-target**	**Mean RT [ms]**	**SD**	**SE**	**CA [%]**	**Prime-target**	**Mean RT [ms]**	**SD**	**SE**	**CA [%]**	**Prime-target**	**Mean RT [ms]**	**SD**	**SE**	**CA [%]**
Experts	c/c	713.91	89.43	21.08	98	m/m	788.21	185.24	46.31	97	m/m	956.52	218.15	65.78	99
Intermediates		777.92	176.8	44.2	97		1,250.27	417.86	104.47	93		984.91	246.7	55.16	96
Novices		1,006.07	360.67	90.17	96		1,757.92	642.44	160.61	85		1,258.77	418.91	76.48	92
Experts	nc/c	769.09	112.08	26.42	96	c/m	808.2	226.5	56.62	98	c/m	1,113.24	280	84.42	98
Intermediates		786.54	181.17	45.29	95		1,231.77	44.46	111.11	94		1,195.25	358.24	80.1	96
Novices		994.55	280.8	70.2	99		1,618.42	584.77	146.19	83		1,760.16	861.27	157.25	95
Experts	c/nc	879.1	130.98	30.87	96	m/c	872.43	186.42	46.61	96	m/c	1,000.54	289.09	87.16	98
Intermediates		972.51	263.69	65.92	97		1,381.72	546.84	136.71	94		1,006.55	242.15	54.15	97
Novices		1,363.24	790.36	197.59	99		1,810.32	765.66	191.41	88		1,248.2	406.88	74.29	91
Experts	nc/nc	825.59	107.16	25.26	98	c/c	885.49	186.71	46.68	97	c/c	1,150.07	353.64	106.63	98
Intermediates		958.21	257.75	64.44	98		1,383.29	574.26	143.56	96		1,242.45	373.93	83.61	97
Novices		1,338.1	530.02	132.5	97		1,827.93	1,034.86	258.71	89		1,705.53	819.9	149.69	95

**Figure 2:**
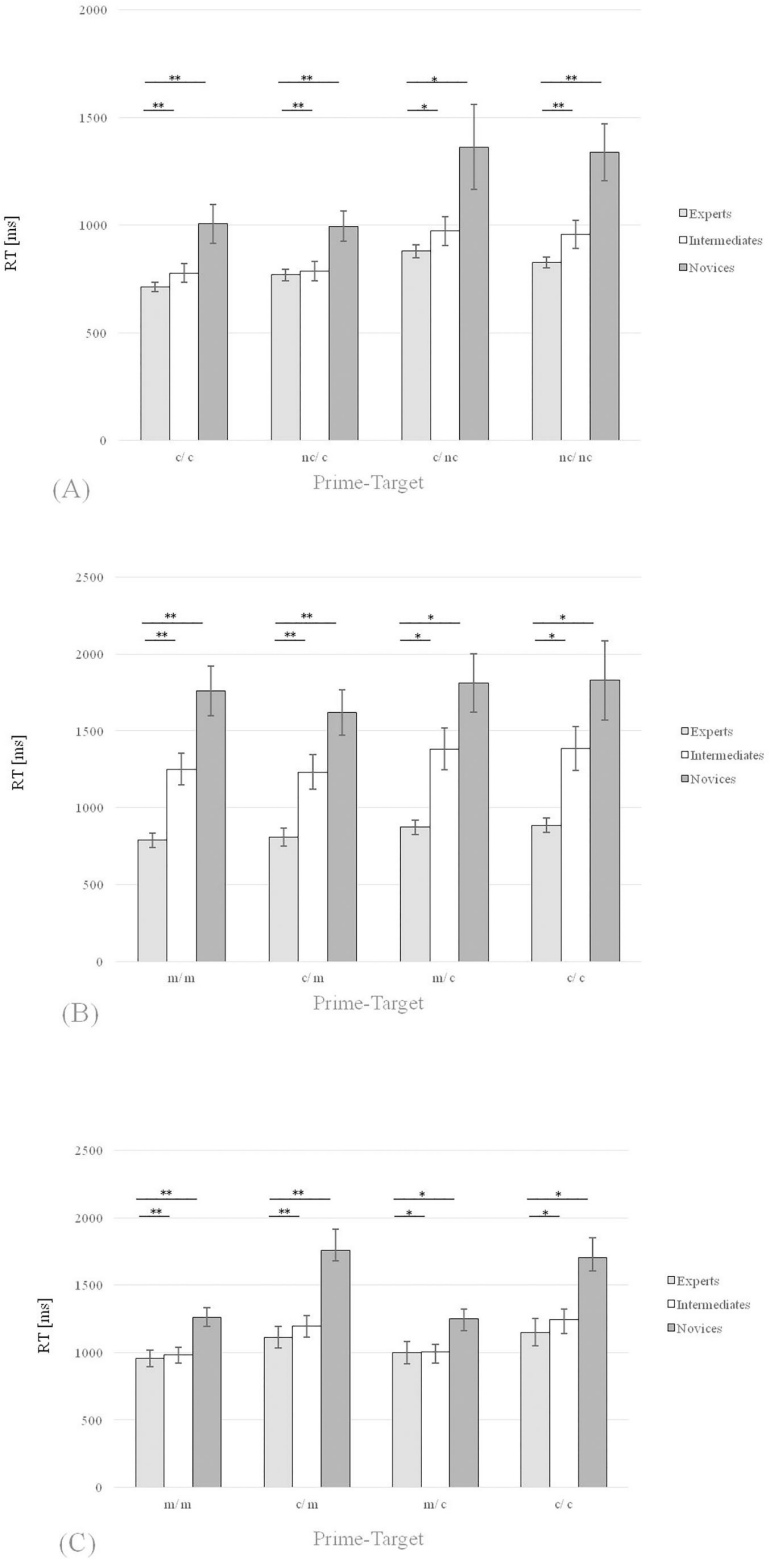
Means of all RTs in Experiments 1 and 2 including the standard errors for all groups and only for the correct answers. **(A)** Experiment 1, setting 1 (*check detection*) **(B)** Experiment 1, setting 2 (*mate detection*) **(C)** Experiment 2 (*planning task, impending check vs. impending mate*). *denotes “significantly” (i.e. *p* < 0.05). **denotes “highly significant” (i.e. *p* < 0.01).

For all significant results Fisher-LSD *post-hoc* were performed and the power (η^2^) was calculated. In a first step, we performed a multivariate analysis (MANOVA) and the results show a significant effect with *F* = 2.88, *p* < 0.05 and with partial η^2^ = 0.22. The one-way ANOVA for the congruent c/c prime-target combination showed a significant effect for the RT with *F*_(2, 44)_ = 6.77, *p* = 0.0027 and η^2^ = 0.24. The *post-hoc* analysis revealed significant differences between *RT*_exp_: *RT*_int_ (*M*_exp_ = 713.91: *M*_int_ = 777.92 ms) and *RT*_exp_: *RT*_nov_ (*M*_exp_ = 713.91: *M*_nov_ = 1,006.07 ms; Figure 2A).

The same analysis for the incongruent nc/c prime-target combination showed a significant effect for the RT with *F*_(2, 44)_ = 6.04, *p* = 0.0048, η^2^ = 0.22. The *post-hoc* comparison showed significant differences between *RT*_exp_: *RT*_int_ (*M*_exp_ = 769.09: *M*_int_ = 786.54 ms) as well as between *RT*_exp_: *RT*_nov_ (*M*_exp_ = 769.09: *M*_nov_ = 994.55 ms; Figure 2A).

Regarding the congruent nc/nc prime-target combination, the analogous analysis showed a significant effect for the RT with *F*_(2, 44)_ = 9.64, *p* = 0.0003, η^2^ = 0.30. The *post-hoc* comparison showed significant differences between *RT*_exp_: *RT*_int_ (*M*_exp_ = 825.59: *M*_int_ = 958.21 ms) as well as between *RT*_exp_: *RT*_nov_ (*M*_exp_ = 825.59: *M*_nov_ = 1,338.10 ms; Figure 2A).

Finally, the same analysis for the incongruent c/nc prime-target combination showed a significant effect for the RT with *F*_(2, 44)_ = 4.51, *p* = 0.0165 and η^2^ = 0.17. The *post-hoc* comparison showed significant differences between *RT*_exp_: *RT*_int_ (*M*_exp_ = 879.10: *M*_int_ = 972.51 ms) as well as between *RT*_exp_: *RT*_nov_ (*M*_exp_ = 879.10: *M*_nov_ = 1,363.24 ms; Figure 2A).

#### Priming effects

Paired *t-*test for depended samples on RT revealed a significant difference on congruency effect only for experts. For experts the comparison of the prime-target pairs c/c and nc/c revealed *t*(16) = 3.21 and *p* = 0.0054. Moreover, the comparison of the prime-target pairs nc/nc and c/nc showed *t*(16) = 3.83 and *p* = 0.0015. The same analysis for the intermediates as well as for the novices revealed no significant differences *t*(14) = 0.41, *p* > 0.05 and *t*(14) = 0.30, *p* > 0.05 for the intermediates and *t*(14) = −0.27, *p* > 0.05 and *t*(14) = 0.29, *p* > 0.05 for the novices.

### Results setting 2

As for our results in setting 1, we performed a multivariate analysis (MANOVA). The results reveal a significant effect with *F* = 4.25, *p* < 0.05 and with partial η^2^ = 0.29. In line with the first experimental setting, we performed a one-way ANOVA taking the number of correct answers (*CA*) as the dependent variable, which showed a significant effect *F*_(2, 44)_ = 5.82, *p* = 0.0057, η^2^ = 0.21. As in setting 1, the number of trials was 80. The *post-hoc* analysis revealed that experts responded correctly significantly more often than novices (*CA*_exp_ = 77.53: *CA*_nov_ = 69.68) and intermediates significantly more often than novices (*CA*_int_ = 75.53: *CA*_nov_ = 69.68), respectively. Further, we performed the same tests for all group comparisons regarding the different prime-target combinations with RT as the dependent variable. Herein, and in the following ANOVAs we considered the RT of the correct responses (performance evidence) for our calculations. Table 1 provides an overview of the descriptive statistics.

The one-way ANOVA for the congruent m/m prime-target combination showed a significant effect for the RT with *F*_(2, 44)_ = 18.80, *p* < 0.001, η^2^ = 0.46. The *post-hoc* analysis revealed significant differences between *RT*_exp_: *RT*_int_ (*M*_exp_ = 788.21: *M*_int_ = 1,250.27 ms) and *RT*_exp_: *RT*_nov_ (*M*_exp_ = 788.21: *M*_nov_ = 1,757.92 ms; Figure 2B).

The analogous analysis for the incongruent c/m prime-target combination showed a significant effect for the RT with *F*_(2, 44)_ = 13.8, *p* < 0.001, η^2^ = 0.39. The *post-hoc* comparison showed significant differences between *RT*_exp_: *RT*_int_ (*M*_exp_ = 808.20: *M*_int_ = 1,231.77 ms) as well as between *RT*_exp_: *RT*_nov_ (*M*_exp_ = 808.20: *M*_nov_ = 1,618.42 ms; Figure 2B).

Moreover, for the congruent c/c prime-target combination the same analysis showed a significant effect for the RT with, *F*_(2, 44)_ = 7.76, *p* = 0.0013, η^2^ = 0.26. The *post-hoc* comparison showed significant differences between *RT*_exp_: *RT*_int_ (*M*_exp_ = 885.49: *M*_int_ = 1,383.29 ms) as well as between *RT*_exp_: *RT*_nov_ (*M*_exp_ = 885.49: *M*_nov_ = 1,827.93 ms; Figure 2B).

The analysis for the incongruent m/c prime-target combination showed a significant effect for the RT with *F*_(2, 44)_ = 11.99, *p* < 0.001, η^2^ = 0.35. The *post-hoc* comparison showed significant differences between *RT*_exp_: *RT*_int_ (*M*_exp_ = 872.43: *M*_int_ = 1,381.72 ms) as well as between *RT*_exp_: *RT*_nov_ (*M*_exp_ = 872.43: *M*_nov_ = 1,810.32 ms; Figure 2B).

#### Congruency effect

A paired *t-*test for dependent samples on RT showed no significant differences among all three groups.

For experts, the comparison of the prime-target pairs m/m and c/m revealed *t*(16) = 0.72 with *p* > 0.05. The comparison of the prime-target pairs c/c and m/c showed *t*(16) = −0.55 with *p* > 0.05. Moreover, for intermediates, the comparison of the prime-target pairs m/m and c/m revealed *t*(14) = −0.24 and *p* > 0.05. In addition, the comparison of the prime-target pairs c/c and m/c showed *t*(14) = −0.04 and *p* > 0.05. Finally, for novices the comparison of the prime-target pairs m/m and c/m revealed *t*(14) = −1.68 and *p* > 0.05; and, the comparison of the prime-target pairs c/c and m/c showed *t*(14) = −0.16 and *p* > 0.05.

## Discussion

In this study, our first goal was to scrutinize chess players' visuocognitive performance by manipulating the prime-target complexity (chessboard size and chess level of threat, i.e., check vs. mate) in two different experimental settings (i.e., check/ mate detection). In order to achieve this, we measured the correctness of the responses and RT among three groups of different proficiency (i.e., experts, intermediates and novices) under special consideration of the priming congruency. For both settings, we hypothesized that experts and intermediates would show significantly more correct responses than novices. No differences were found concerning the amount of the correct responses among all groups in setting 1. However, in setting 2 experts responded significantly more accurately compared to intermediates and novices. Further, intermediates provided a better performance of correctness than novices.

According to the existing findings, we assumed that experts would show a significantly shorter RT than the other, less skilled groups. Finally, we expected to reveal a congruency-priming-effect for experts. The results confirm existing findings of Kiesel et al. (2009) and partly our assumption that expertise is consistent to the information processing rate but limited through the prime-target complexity. Regarding the between-group differences for RT, we were able to show that experts responded significantly faster than intermediates and novices in both settings (complexity manipulation in a static constellation). These performance differences were also measured for both conditions (congruent/incongruent). Hence, experts provide a superior visuocognitive performance.

The analysis of the priming effects revealed a congruency effect only in setting 1 for the experts. In setting 2 we revealed no congruency effects at all. We assume that these results can be explained on the one hand with the *chunking model* (Chase and Simon, 1973a,b) and on the other hand through the higher prime-target complexity. In order to examine the referred limitations, the conduction of a further experiment appears essential, whereas the reasoning as well as the reaction planning require a more comprehensive visuocognitive performance.

## Experiment 2: Subliminal priming and planning the next move

In the second experiment we also followed up on the question of whether the differences between the participants' expertise affects the results of the studies of Kiesel et al. (2009) and Sheridan and Reingold (2017). Therefore, we compared experts, intermediates, and novices with respect to their visuocognitive performance in a more complex reaction time task than in Experiment 1. In order to realize the mentioned complexity, we combined the approaches of Kiesel et al. (2009) and Sheridan and Reingold (2017). As in Experiment 1 visual chess stimuli were presented on a computer screen. In contrast to Experiment 1, there was only one setting, and the task was to *plan the next move* (mating or checking the king) (Figure 1D) while the prime stimuli (Figure 1C) showed a static situation (king is already mated or checked).

### Complexity manipulation

In contrast to Sheridan and Reingold (2017), we did not use a knight, which has a considerably complex movement pattern. Instead, each stimulus contained a black king, two black pawns and a white attacker (in half of the stimuli a queen and in the other half a rook). Both queen and rook have linear movement patterns in contrast to the knight. Unlike the Sheridan and Reingold (2017) design, only one prime-target stimulus was presented per trial (e.g., Experiment 1). The scenario for each target stimulus was: “White has to move - can the black king be mated within one move?”. In this sense, the response targets require future planning similar to Sheridan and Reingold (2017). An impending mate must be identified. Therefore, the results of the movements in our stimuli were more complex and included a discrimination task (“Is there a mate or check impending?”).

We introduced prime stimuli preceding the target stimuli similar to the experimental course in Experiment 1. Each prime stimulus can be either congruent or incongruent with the result of the move in the subsequent target stimulus. Hereby, congruency was defined analogously to Experiment 1. Prime and target stimulus showed the same condition (e.g., prime stimulus with a mate and target stimulus with an impending mate).

We hypothesized that experts would perform significantly better regarding the number of the correct responses and the RT and would show congruency effects compared to intermediates. Following up, we assumed that intermediates would have a shorter RT than novices. We expected no congruency effects at all for the two last groups.

### Materials and methods

#### Design

Experiment 2 of the present study is also based on a 3 × 2 design (expertise × prime congruency) having RT as the dependent variable. In contrast to Experiment 1, only one setting and one type of task is employed: “Can the king be mated within the next move, or can it be only checked?”.

#### Equipment

In order to realize the present study, the same apparatus as in Experiment 1 was used. Further, the selected stimuli were provided using the Presentation^®^ software (Neurobehavioral Systems, 2019). Participants' RTs were registered by an external button box.

#### Participants

A total of 61 participants (*M*_age_ = 28.51, *SD* = 11.60) passed the test trial in which they had to prove that they know the pieces used in this experiment and that they are able to distinguish mate and mere check. We classified *N*_nov_ = 30 novices, *N*_int_ = 20 intermediates, and *N*_exp_ = 11 experts. Herein, the criteria for chess expertise classification (i.e., ELO/ DWZ ranking), are the same as in Experiment 1 and all novices proved their knowledge about chess rules, pieces' movement patterns, and checking and mating situations to be sufficient. None of the participants took part in the first experiment. Prior to the data acquisition all participants have been informed about the purpose of the study and gave informed consent.

#### Stimuli and procedure

We employed a total of 24 stimuli of a 4 × 4 chessboard (45 mm square) representing the upper left quarter of a chessboard. As in Experiment 1, the black king is always located in the upper left corner. Twelve chess constellations were used as targets and the other 12 as primes. Six targets showed an impending mate (analogously to the notation in Experiment 1 denoted by “*m*”). The constellation implied a queen (three stimuli) or a rook (three stimuli) attacking the king. In contrast, the other six targets presented an impending check (denoted by “*c*”), including a queen (three stimuli) or a rook (three stimuli). Six of the prime stimuli presented the king already mated (no move to be planned). The other six prime stimuli contained a mere check. Due to the lack of combinatorial possibilities for a rook checkmating the king on a 4 × 4 chessboard, only two of them employ a rook. In the six constellations showing a mere check both queen and rook were employed three times.

Each trial included a fixation cross (400 ms), a blank sequence (70 ms), the prime stimulus (300 ms), another blank screen (70 ms), and finally the target. We took into account that the complexity of our stimuli again has been increased and this requires more intense information processing than in Experiment 1. Therefore, we used a longer prime presentation time of 300 ms. Again, this is under the threshold of 500 ms which is generally considered to imply conscious perception (Elgendi et al., 2018). Five seconds were allowed as the maximum response time. Two blank screens were introduced to diminish the awareness of the primes. Participants were instructed analogously to Experiment 1 and were asked to give their decision *via* the button box. The experiment consisted of two blocks, each of them containing 144 prime-target-pairs, i.e., 12 (primes) times 12 (targets), presented in a random order. In line to the first experiment and in contrast to Kiesel et al. (2009), participants received no information about the structure of the prime stimuli. There was no prime detection task as we did not employ target primes and therefore did not have to distinguish between novel and target primes.

## Results

We hypothesized that experts have a significantly better RT and congruency effects than intermediates. Intermediates in turn will have a better RT than novices whom we expect not to reveal a congruency effect at all.

As for all our results in Experiment 1, we performed a multivariate analysis (MANOVA) and the results show a significant effect with *F* = 2.38, *p* < 0.05 and with partial η^2^ = 0.148.

### Reaction time

For all group comparisons regarding the different prime-target combinations we performed a one-way ANOVA having RT as the dependent variable. For all significant results Fisher-LSD *post-hoc* were performed and the power (η^2^) was calculated. We took into account only the RT of the correct responses (performance evidence) for our calculations (for an overview of the descriptive statistics see Table 1).

As in Experiment 1, we took the number of correct answers as evidence of performance and performed a one-way ANOVA which showed a significant effect regarding the quality of registered answers: *F*_(2, 58)_ = 5.97, *p* = 0.004, η^2^ = 0.17. The number of trials was 288. The *post-hoc* analysis revealed significantly more correct responses for experts than for novices (*CA*_exp_ = 282.18: *CA*_nov_ = 268.73) as well as for the intermediates compared to the novices (*CA*_int_ = 277.35: *CA*_nov_ = 268.73).

The one-way ANOVA for the congruent m/m prime-target combination showed a significant effect for the RT with *F*_(2, 58)_ = 5.33, *p* = 0.0075, η^2^ = 0.16. The *post-hoc* analysis revealed significant differences between *RT*_exp_: *RT*_int_ (*M*_exp_ = 956.52: *M*_int_ = 984.91 ms) and *RT*_exp_: *RT*_nov_ (*M*_exp_ = 956.52: *M*_nov_ = 1,258.77 ms; Figure 2C).

The same analysis for the incongruent c/m prime-target combination showed a significant effect for the RT with *F*_(2, 58)_ = 6.36, *p* = 0.0032, η^2^ = 0.18. The *post-hoc* comparison showed significant differences between *RT*_exp_: *RT*_int_ (*M*_exp_ = 1,113.24: *M*_int_ = 1,195.25 ms) as well as between *RT*_exp_: *RT*_nov_ (*M*_exp_ = 1,113.24: *M*_nov_ = 1,760.16 ms; Figure 2C).

The congruent c/c prime-target combination analysis revealed a significant effect for the RT with *F*_(2, 58)_ = 4.72, *p* = 0.0126, η^2^ = 0.14. The *post-hoc* comparison showed significant differences between *RT*_exp:_ *RT*_int_ (*M*_exp_ = 1,150.07: *M*_int_ = 1,242.45 ms) as well as between *RT*_exp_: *RT*_nov_ (*M*_exp_ = 1,150.07: *M*_nov_ = 1,705.53 ms; Figure 2C).

Finally, the ANOVA for the incongruent m/c prime-target combination showed a significant effect for the RT with *F*_(2, 58)_ = 3.89, *p* = 0.0259, η^2^ = 0.12. The *post-hoc* comparison showed significant differences between *RT*_exp_: *RT*_int_ (*M*_exp_ = 1,000.54: *M*_int_ = 1,006.55 ms) as well as between *RT*_exp_: *RT*_nov_ (*M*_exp_ = 1,000.54: *M*_nov_ = 1,248.20 ms; Figure 2C).

#### Congruency effect

As in experiment 1, we performed paired *t-*tests having RT as the dependent variable, taking into account only the correct answers. Herein, for all expertise groups we observed a significant *positive* congruency effect for the prime-target pair comparison m/m vs. c/m and a significant *negative* congruency effect for the prime-target pair comparison c/c vs. m/c.

For the experts, the comparison of the prime-target pairs m/m and c/m revealed significant differences regarding the RT with *t*(10) = 5.02, *p* < 0.001. Moreover, the comparison of the prime-target pairs c/c and m/c for the same group showed *t*(10) = −4.18, *p* = 0.002.

Furthermore, the paired *t*-test for the intermediates and the prime-target pairs m/m and c/m revealed a significantly faster response by the congruent condition with *t*(19) = 5.41 and *p* < 0.001. The comparison of the prime-target pairs c/c and m/c showed faster response by the incongruent pairs with *t*(19) = −5.54 and *p* < 0.001.

Finally, the comparison of novices' RT for the prime-target pairs m/m and c/m revealed *t*(29) = 4.7 and *p* < 0.001. The comparison of the prime-target pairs c/c and m/c showed that novices react faster when the prime-target pairs were incongruent with *t*(29) = −4.47 and *p* < 0.001.

## Discussion

In this study we have supplied a priming paradigm in a reaction time task utilizing stimuli with instantaneous and static chess positions (i.e., check or no check). In order to expose visuocognitive processing limitations related to the prime-target complexity (static vs. dynamic situation and level of chess threat, i.e., check vs. mate), we conducted the second experiment, increasing the target detection processing for the participants by using a dynamic scenario of thinking ahead and planning the next move. In this context, the participants had to decide if “The king can be checked but not mated in one move”. The stimuli (c/m, m/m, m/c, and c/c) were presented to the three participating groups in a randomized order. The reaction time and the correctness of the answers were registered and used for the further statistical analysis under special consideration of the priming congruency (a total amount of 288 responses for each participant).

We first hypothesized that expertise significantly affects the responses' correctness and second, following the chunking theory, we expected that the RT will be also significantly related to the expertise level. However, we assumed that the increased target complexity will lead to a limitation of the priming effects. Regarding the response correctness, the analysis revealed that experts responded more accurately than the intermediates as well as the novices. The comparison of the RT for all prime-target conditions (congruent and incongruent) showed that experts reacted significantly faster compared to the other two groups. Hence, for the stimuli selected in Experiment 2 (more chess pieces on a larger chess board) experts' response performance is not affected. This supports the novice-expert differences in Charness et al. (2001), Reingold et al. (2001), and Sheridan and Reingold (2014) by indicating that experts benefit from a superior visuocognitive performance (Kiesel et al., 2009; Sheridan and Reingold, 2017).

The analysis of the priming effects showed that all groups reacted faster by the mate congruent prime-target combination (m/m compared to c/m). In contrast, for the check detection task all groups reacted faster when prime and target were incongruent. This means that our hypothesis that the planning task would limit visuocognitive performance (e.g., priming effects) of the participants can be rejected. However, the observed reversed priming effects (Eimer and Schlaghecken, 2002) are of considerable interest. For example, Kahan (2000) and Goodhew et al. (2011) associate reversed priming effects as no priming with incorrect prime identification. Therefore, the high-threat detection task (a mate, which means the end of the game) in the prime stimuli in combination with the conscious target analysis can not only cause but also explain these effects.

## General discussion

In the present study, we have investigated chess players' visuocognitive performance as a function of expertise and degree of complexity in decision-making tasks. Herein, we have manipulated the prime-target complexity with respect to the chessboard size, the chess threat level and by either judging static situations or planning the next move. In summary, we have hypothesized that expertise has a significant impact on the correctness of response as well as on the RT. Finally, we expected priming effects only for the experts (Kiesel et al., 2009) and that these effects vanish with increasing prime-target complexity starting with a static chess situation switching to a dynamic task which implies the planning of the next move. Our results confirm most of our hypotheses.

In Experiment 1, we have shown that highly-skilled chess players benefit from superior visuocognition and reveal a faster response by decision making which supports past findings (Charness et al., 2001; Reingold et al., 2001; Sheridan and Reingold, 2014). In the *check detection* setting experts reacted faster but not more accurately as the other groups. The response priming effects which were observed only for the experts indicate that they are able to take advantage of acquired perceptual chunks (Chase and Simon, 1973a) which is also in line with the results of Kiesel et al. (2009). Similar results were found in the second setting with regard to the response accuracy. Experts performed not only faster but also gave significantly more correct answers. Furthermore, the lack of priming effects in the *mate detection* setting contributes additional insights regarding the findings of Kiesel et al. (2009) and especially concerning limitations of experts' advantages for acquired perceptual chunks. Addressing the non-significant priming effect in the second setting, we argue that the detection of a *mate* is more complex than the one of a *check* and requires conscious comparisons processing and exhaustive search.

In Experiment 2, we have confirmed that expertise is advantageous regarding the accuracy and the reaction time of planning the next move (detection of *mate in one*). Experts and intermediates showed a significantly higher processing efficiency than novices reacting significantly more accurately. Additionally, experts were significantly faster in all experimental conditions compared to the other groups. This is in line with results of Charness et al. (2001), Kiesel et al. (2009) and Sheridan and Reingold (2014) and Sheridan and Reingold (2017). Finally, not only experts but also all groups showed a congruency effect. That is, here we could not find any evidence about limitations of visuocognitive performance, which are related to a high prime-target complexity.

These results can be explained by the longer presentation of the complex prime stimulus, which includes a mate identification task, triggers the reversed priming effect, and probably in our case was increased through the searching of the higher threat for the king (mate).

## Limitations

We are aware that our study has several limitations. First, accordingly to the fact that we use only two fixed prime durations, it is possible that the results do not reveal all fine differences according to the visuocognitive performance among the three different expertise groups. Second, we have increased the complexity of the stimuli and task quite coarsely (i.e., from a mere check identification task in static positions in Experiment 1, setting 1 to a mate identification task in Experiment 1, setting 2 and furthermore up to planning the next move in Experiment 2). We propose that a finer grading of complexity concerning stimuli and task combinations could lead to a more detailed mapping of the visuocognitive performance of chess players with different levels of expertise.

Research must invest more effort using a multimodal approach (visual strategies – eye tracking, sensor technologies, and usage of real chess boards for a realistic 3D chess scenario) in order to clarify the insights about the processes behind the visuocognitive performance of chess players. Such an approach will allow detailed insights into players' visuocognition and will deliver an amount of additional information, which can extend and specify the research as well as for instance the application of virtual chess players.

## Conclusions

Altogether, our results indicate that high processing efficiency and a superior visuocognitive performance (correctness of answers, RT) requires a high level of expertise. Nevertheless, visuocognitive performance regarding priming effects is not only affected by the manipulation of the prime-target complexity but probably also by the prime duration. We assume that the potential of threat (mate or check) presented in the target also affects the priming effects (i.e., in a check situation participants have to perform more than one comparison before they make their decision and correctly rule out a mate). In this sense of reasoning, our findings facilitate deeper insights into congruency effects and give impulses for future research concerning interrelations between congruency effects and planning of the next move in given chess constellations.

## Data availability statement

The raw data supporting the conclusions of this article will be made available by the authors, without undue reservation.

## Ethics statement

The studies involving human participants were reviewed and approved by German Research Foundation (DFG) Ethics Commission of Bielefeld Sport Sciences Department. The participants provided their written informed consent to participate in this study.

## Author contributions

TK, KE, DK, and TS contributed to conception and design of the study. TK organized the implementation of the study and wrote the first draft of the manuscript. TK and KV performed the statistical analysis and wrote sections of the manuscript. All authors contributed to manuscript revision, read, and approved the submitted version.

## Funding

This research was funded by the German Research Foundation (DFG) in the frame of the CEEGE project. The funding agency was not involved in the study design, collection, analysis, or interpretation of the data, writing of the report, nor in the decision to submit the article for publication.

## Conflict of interest

The authors declare that the research was conducted in the absence of any commercial or financial relationships that could be construed as a potential conflict of interest.

## Publisher's note

All claims expressed in this article are solely those of the authors and do not necessarily represent those of their affiliated organizations, or those of the publisher, the editors and the reviewers. Any product that may be evaluated in this article, or claim that may be made by its manufacturer, is not guaranteed or endorsed by the publisher.
